# Primary Spinal Cord Glioblastoma

**DOI:** 10.7759/cureus.18464

**Published:** 2021-10-03

**Authors:** Elizabeth Escobar Peralta, Laura Hernández Sánchez

**Affiliations:** 1 Department of Radiotherapy, Hospital Juaréz de México, Ciudad de México, MEX; 2 Department of Radioneurosurgery, Institute of Neurology and Neurosurgery, Ciudad de México, MEX

**Keywords:** surgery, ependymoma, glioblastoma, primary spinal tumor, radiotherapy

## Abstract

We present the case of a 29-year-old patient whose pain began with the interscapular region, progressing to paresthesia and loss of muscle strength in the lower extremities. MRI of the spine was done, a lesion was found in T2 to T6, ependymoma was suspected and was taken to subtotal resection with laminectomy, the histopathological report, as well as the immunohistochemistry, was compatible with glioblastoma type not otherwise specified (NOS). He received adjuvant with radiotherapy and concomitant chemotherapy, but he progressed to the cervical and lumbar spine, the patient died 16 months after diagnosis. A review of the literature is made and the clinical and radiological characteristics and treatment protocols that have been used in this entity are reported.

## Introduction

Primary spinal cord glioblastoma multiforme (GBM) is a CNS tumor that is clinically, histologically, and genetically heterogeneous, and is a rare tumor type accounting for 1% to 5% of all glioblastomas and only 1.5% of all tumors of the spinal cord [1-3].

This tumor occurs particularly in younger patients (mean: 26 years old), is a little more common in men [1,4], and chiefly occurs in the cervical and thoracic spine [1,5]. Its clinical manifestations depend on the location and extent of the disease [3]. MRI and histopathological examination are indispensable for establishing the diagnosis [6-7]. The most recommended treatment is similar to intracranial glioblastoma that includes surgery, radiotherapy and chemotherapy, also with poor clinical results.

Due to its low incidence, the literature is limited to reports of cases or small series, so it is not surprising that little is known about the clinical characteristics and the treatment.

## Case presentation

**Figure 1:**
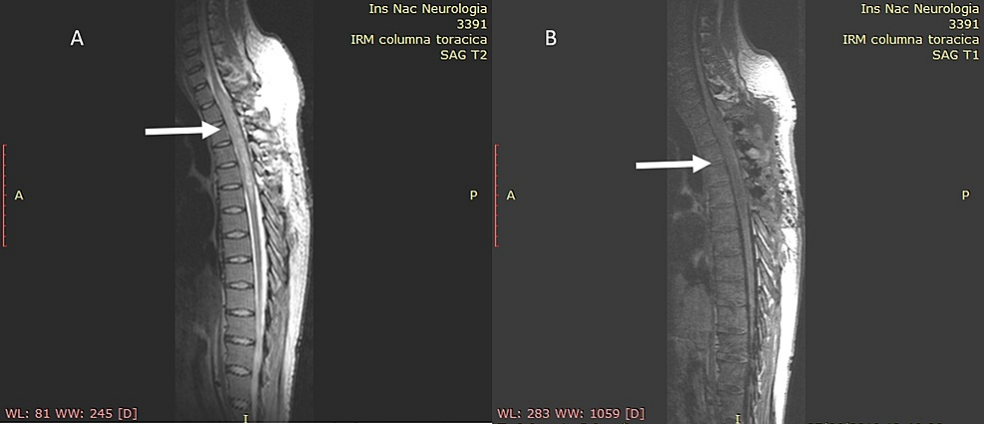
MRI spine, sagittal view showing (arrows) a lesion at T2-T6. A) T2- and B) T1-weighted images.

A 29-year-old male patient, with no relevant medical or family history, experienced mild dorsal pain, paresthesia, and loss of muscle strength in the right leg, and three months later presented the same symptomatology in the contralateral leg, as well as urinary incontinence. Two months later he had numbness in both lower limbs and could not walk as well as constipation; non-contrast and contrast-enhanced MRI of the spine revealed a hypointense and hyperintense lesion from T2 to T6 vertebrae on T1 to T2 images, respectively (Figure 1).

An extended MRI of the brain and total spine showed no evidence of dissemination. A probable ependymoma was considered and laminectomy with near-total tumor resection was performed, the pathological analysis revealed glioblastoma, WHO grade-IV, due to the rarity of the diagnosis, another pathological examination with immunohistochemical was performed: synaptophysin negative, S-100 positive, glial fibrillary acidic protein (GFAP) positive, so, the diagnosis of glioblastoma type not otherwise specified (NOS) was confirmed.

**Figure 2:**
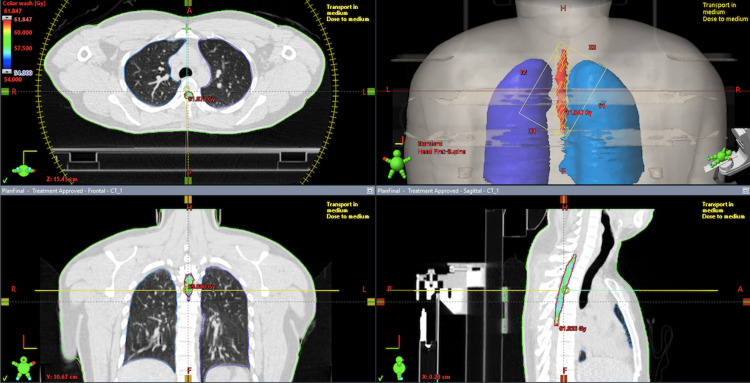
Radiation treatment plan showing target volume in red.

We offered adjuvant concurrent chemoradiation (CCRT). A total radiation dose of 5400 cGy in 30 fractions at 180 cGy per fraction per day was given to the tumor bed along (from T2 to T6) with concomitant temozolomide at a dose of 75 mg/m2 (Figure 2).

Then, the patient received adjuvant chemotherapy (QT) with seven cycles of carboplatin and irinotecan as a protocol due to a shortage of temozolomide in our institution.

**Figure 3:**
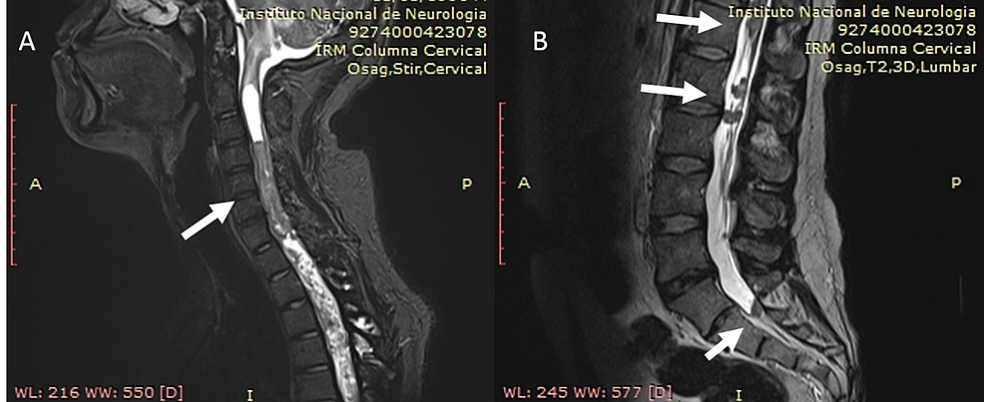
A) cranial and B) lumbar MRI showing multiple implants.

Three months after the completion of maintenance QT, the patient complained of pain in the lower back, and we repeated MRI of the craniospinal axis. Many implants were observed from C4 to T8 and in the lumbar region, nodular images were found next to the nerve roots (Figure 3).

No lesions were found in the brain parenchyma, but we considered leptomeningeal spread, so, a total radiation dose of 3000 cGy in 10 fractions at 200 cGy per fraction per day was given to the whole brain and total spine with volumetric modulated arc therapy (VMAT) technique, as palliative treatment, unfortunately, he presented Collet-Sicard syndrome and died a week later.

## Discussion

Primary tumors of the spinal cord represent a heterogeneous group of diseases in the CNS, their treatment is very complex owing to their low incidence and morbidity associated with treatment. These tumors are divided into three anatomical categories: intradural intramedullary (astrocytoma, ependymoma, glioblastoma, hemangioblastoma); intradural extramedullary (myxopapillary ependymoma, meningioma, neurofibroma, schwannoma); and extradural (chordoma, chondrosarcomas), hematopoietic malignancies [8].

In adults, 80% of neoplastic lesions located in the spinal cord are extramedullary tumors. Low-grade gliomas account for 50-88% of intramedullary tumors [9]. Until 2018, fewer than 200 cases of intramedullary GBM have been reported in the literature [10-11]. Unlike its intracranial counterpart, the primary spinal cord GBM has a predilection between the second and third decade of life [12], with a mean of 26 years and men are more commonly affected than women (57% vs 43%) [1,4], in this case reported, the patient was 29 years old male. It occurs usually in the cervicothoracic region in up to 70% of the cases, followed by the lumbar area with 9.7% and less frequent in the medullary cone with 5.5% [1-2, 5, 13-15]. Tumors of the thoracic region have a better prognosis [16]. Their clinical characteristics depend on the location and extent of the spinal cord, and the most common reported symptom is pain. Our patient had a three-month history of pain, numbness, and progressive decrease of muscle strength in extremities [3]. MRI is considered the gold standard imaging technique to diagnose primary spinal cord GBM, it shows T2 hyperintense infiltrating lesions with heterogeneous enhancement after contrast material injection in T1-weighted sequences; differential diagnosis with other intramedullary tumors is difficult [6-7, 17], so the definitive diagnosis is determined with a pathological examination, in this case ependymoma was considered.

Despite aggressive treatment, the prognosis of patients with primary spinal cord GBM remains poor, with an overall survival of 10-14 months [4, 18], in the case presented, the overall survival was 16 months. Konar et al., in 2015, presented an analysis of factors that determine the final outcome in primary spinal cord GBM and showed that patients between 18 and 65 years of age had a better overall survival (14 months) compared with those of extreme age outside that range (< 18 years, 10.5 months and > 65 years, 2 months; log rank p 0.0005) and with surgery followed by adjuvant therapy (radiotherapy, chemotherapy, or both) was significantly associated with improved survival (p 0.0005) [11].

Therapeutic options for primary spinal cord GBM remain controversial because there are few reported cases, but usually include surgery, radiation therapy (RT), and QT similar to intracranial GBM. Surgical options include gross total resection (GTR), subtotal resection (STR), and biopsy. Complete resection with the greatest preservation of neurological function is the most recommended surgical approach, however, this pathology tends to be infiltrative and it is difficult to distinguish between the margin of the tumor and the adjacent normal tissue, the association with survival according to the type of resection has not yet been established, there are studies that do not find benefit with a GTR in terms of overall survival and even associate it with higher mortality [1, 11, 16].

Adjuvant radiotherapy is recommended because there is a high probability of spread and recurrence, the ideal dose has not been established, in the literature, doses of 50.4 Gy with concomitant temozolomide at a dose of 75 mg/m^2^ similar to intracranial glioblastoma are reported [19], after completion of CCRT, maintenance chemotherapy with temozolomide at a dose of 150 mg/m^2^ is continued, but the results in the primary spinal cord GBM are still not clear and it is important to mention that we have dose-limiting toxicity in spinal cord because of its radiosensibility [2]. In conventional fractionation, it has been reported that the tolerance dose in lengths of 5 to 10 cm is 50 Gy and 47 Gy for lengths of 20 cm because the probability of myelopathy is less than 5% to 5 years, for this reason, a maximum dose of 45-50 Gy is acceptable [20]. In this case, the patient received 54 Gy to the primary tumor because we considered the radioresistance to radiotherapy of the GBM.

After four months without neurological deterioration, our patient presented leptomeningeal dissemination because MRI of the whole brain and spine revealed new lesions in the cervical and lumbar spine. This probability of leptomeningeal dissemination has already been reported in the literature, the craniospinal irradiation has even been proposed, but, its effectiveness remains unclear [11]. In this case, we decided the craniospinal irradiation as a palliative treatment, unfortunately, the patient died at the end of this treatment. Intrathecal CT has shown a survival benefit (2-4 months) when leptomeningeal dissemination occurs, nevertheless, it has a significant risk of producing myelitis and myelosuppression [15].

All current therapeutic measures have produced disappointing results and little data is available on their true value.

## Conclusions

The initial presentation and evolution of this case were like the cases reported in the literature, for example, the age of 26 years, thoracic location; the diagnosis was made with histopathological examination, even a second evaluation was needed because of the rarity of the pathology. It had clinical progression despite aggressive management (surgery, radiotherapy, and concomitant chemotherapy), unfortunately in our institution, there was a shortage of temozolomide, so treatment with carboplatin and irinotecan was chosen, but the patient presented leptomeningeal dissemination and was treated with craniospinal irradiation. The patient had a survival of 16 months (a little longer than that reported in the literature). All this shows that spinal GBM is an aggressive pathology, with an unfavorable prognosis despite treatments.

There are no clinical studies that regulate the therapeutic conduct to be followed, for this reason, and we consider that it is important to improve the registration and cooperation of many centers to understand this pathology.
